# Advanced Functions Embedded in the Second Version of Database, Global Evaluation of SARS-CoV-2/hCoV-19 Sequences 2

**DOI:** 10.3389/fmed.2022.813964

**Published:** 2022-04-11

**Authors:** Kailing Li, Audrey K. Y. Wang, Sheng Liu, Shuyi Fang, Alex Z. Lu, Jikui Shen, Lei Yang, Chang-Deng Hu, Kai Yang, Jun Wan

**Affiliations:** ^1^Department of BioHealth Informatics, Indiana University School of Informatics and Computing, Indiana University-Purdue University Indianapolis, Indianapolis, IN, United States; ^2^Park Tudor School, Indianapolis, IN, United States; ^3^Department of Medical and Molecular Genetics, Indiana University School of Medicine, Indianapolis, IN, United States; ^4^Collaborative Core for Cancer Bioinformatics (C^3^B) shared by Indiana University Simon Comprehensive Cancer Center and Purdue University Center for Cancer Research, West Lafayette, IN, United States; ^5^The Wilmer Eye Institute, Johns Hopkins University School of Medicine, Baltimore, MD, United States; ^6^Herman B Wells Center for Pediatric Research, Department of Pediatrics, Indiana University School of Medicine, Indianapolis, IN, United States; ^7^Department of Medicinal Chemistry and Molecular Pharmacology, Purdue University, West Lafayette, IN, United States; ^8^Purdue University Center for Cancer Research, Purdue University, West Lafayette, IN, United States; ^9^The Center for Computational Biology and Bioinformatics, Indiana University School of Medicine, Indianapolis, IN, United States

**Keywords:** COVID-19, SARS-CoV-2, SNV, lineage, enrichment, database, newly emerging

## Abstract

The Global Evaluation of SARS-CoV-2/hCoV-19 Sequences 2 (GESS v2 https://shiny.ph.iu.edu/GESS_v2/) is an updated version of GESS, which has offered a handy query platform to analyze single-nucleotide variants (SNVs) on millions of high coverages and high-quality severe acute respiratory syndrome coronavirus 2 (SARS-CoV-2) complete genomes provided by the Global Initiative on Sharing Avian Influenza Data (GISAID). Including the tools in the first version, the GESS v2 is embedded with new functions, which allow users to search SNVs, given the viral nucleotide or amino acid sequence. The GESS v2 helps users to identify SNVs or SARS-CoV-2 lineages enriched in countries of user’s interest and show the migration path of a selected lineage on a world map during specific time periods chosen by the users. In addition, the GESS v2 can recognize the dynamic variations of newly emerging SNVs in each month to help users monitor SNVs, which will potentially become dominant soon. More importantly, multiple sets of analyzed results about SNVs can be downloaded directly from the GESS v2 by which users can conduct their own independent research. With these significant updates, the GESS v2 will continue to serve as a public open platform for researchers to explore SARS-CoV-2 evolutionary patterns from the perspectives of the prevalence and impact of SNVs.

## Introduction

In December 2019, the first-ever human coronavirus disease 2019 (COVID-19) case was found in Wuhan, China. This disease was found to be caused by the novel coronavirus called the “severe acute respiratory syndrome coronavirus 2 (SARS-CoV-2)” (1). For the past 19 years, there have already existed two emergences caused by coronavirus SARS (2002–2003) and the Middle East respiratory syndrome (MERS, from 2012 to till present). This time, the outbreak of SARS-CoV-2 has been causing a huge socioeconomic problem all over the world with more critical challenges to the research, public health, and medical communities (2). As of January 21, 2022, over 350 million cases of COVID-19 with about 5 million deaths have occurred globally. In a rapid response to the pandemic, the vaccines have been successfully developed followed by broad clinical trials. Several vaccines were finally approved by the Food and Drug Administration (FDA) with the authorization of emergence uses and by WHO and other countries or organizations. More than a billion vaccine shots have been distributed to a large population, but it is still urgent to explore and understand the evolution and transmission of SARS-CoV-2 to make correct public health policy.

At present, over 7 million SARS-CoV-2 viral genome sequences have been submitted and made public *via* the well-known online platform, the Global Initiative on Sharing Avian Influenza Data (the GISAID).^1^ The huge amount of available data unlock the potentials to unveil the evolution of SARS-CoV-2, even though retrieving the useful information from the database becomes a big challenge. The GISAID itself incorporates various tools for the analyses on SARS-CoV-2-related functions (3, 4). The platform implements data searching, downloading, and hCoV-19 submission/variants tracking functions. But, the function of variants tracking in the GISAID can only show the cumulative numbers of the variants, given a timeframe, without providing a clue about the exact country/area where the variants/lineage migrated from. There also exist many other COVID-19 databases. For example, users can check SARS-CoV-2 variants and mutations on the “Coronavirus antiviral and resistance database”^2^ (5) from Stanford and the “outbreak.info”^3^ (6) by Scripps Research. Users can utilize these databases to view COVID-19 trends and explore the SARS-CoV-2 lineage and mutation information, while searching research papers related to COVID-19 or SARS-CoV-2. The “Nextstrain”^4^ (7) is another web tool that can conduct a viral evolution analysis on COVID-19 and others such as influenza. For example, they can assign a viral sequence to the corresponding SARS-CoV-2 clade and locate it on the SARS-CoV-2 tree by comparing the sequence against the SARS-CoV-2 reference genome. From animated plots, users can learn the locations where the clades were born and know the proportion of each variant in each geographical area. However, their global visualization has a limitation on the sample numbers. The Pango nomenclature from the “COV-Lineages”^5^ (8) is used to show the dynamic changes of SARS-CoV-2 lineages in terms of occurrences in different countries at different time points. It has several online tools for sequence analysis and visualization, such as “Pangolin” that assigns the most likely lineage to SARS-CoV-2 sequences. It also provides more information about the sub-lineages and variants of concern reports. Some other platforms allow users to upload their own SARS-CoV-2 sequences to identify the alterations online, such as the “COV-GLUE,”^6^ which is still in the development phase and hopefully can help users recognize amino acid replacements, insertions, and deletions.

People have put efforts to develop databases regarding vaccine designs and targets for the COVID-19. For example, “COVIDep”^7^ (9) is devoted to vaccine target recommendations based on the data from the GISAID and Virus Pathogen Resource (VIPR) databases (10). This platform provides overall patterns of SARS-CoV-2 sequences, e.g., geographical distributions, temporal distribution, and temporal distribution on specific locations/countries, regardless of the sequence mutations. They can judge the epitopes by the genetic matching scores with the SARS-CoV-2 consensus sequence in the targeting region while considering the sequence conservations.

There is no doubt that web-based databases introduced above and others unmentioned in this study are very useful from their own perspectives. However, to our knowledge, most SARS-CoV-2 databases focus on the lineages and/or amino acid changes on specific variants without more detailed information about single-nucleotide variants (SNVs) at the level of the single nucleotide. Hence, we developed the Global Evaluation of SARS-CoV-2/hCoV-19 Sequences (GESS) (11), which analyzes the variations in both nucleic acid and amino acid levels on millions of high-quality complete SARS-CoV-2 genomes. The GESS allows users to browse and search individual SNVs based on viral genome positions or regions of interest. It provides users with detailed information about SARS-CoV-2 SNVs, including the time and locations the SNVs were first time identified, distinct dynamic transmission and evolution patterns for SNVs in different countries/areas, and co-occurrence relationship among SNVs. The embedded tools can assist users in performing diverse functional analyses. Results can be downloaded directly from the website as well. Since it was made public in October 2020, the GESS has attracted extensive attention. According to a Google analysis tool, the GESS has been visited 9,191 times from 5,437 IP addresses in 116 countries as of January 21, 2022. In this study, we presented the advanced version of GESS, named GESS v2, with multiple improvements in the older version and more newly developed functions.

The GESS v2 analyzes over 4 million high-coverage SARS-CoV-2 complete genome sequences as of January 21, 2022. Due to the increasing volume of the sequencing data, the processing method for the older version of GESS made the database loading very slow. In GESS v2, we have saved precalculating results and optimized the data format by truncating results into multiple small data blocks, which can reduce the loading time significantly. We have also improved the designs for user interface (UI) and user experience (UX) including, but not limited to, changing the font size and background color to make GESS v2 more efficient and user-friendly. The bootstrap4 framework (12) has been adopted in the GESS v2 so that the web-based platform can be recognized by different devices/operating systems and its layout of contents can be automatically adjusted to fit the device. Based on users’ feedbacks, a table has been added in the “Summary Info” on the “Home” page to show monthly numbers of SARS-CoV-2 genomes collected in each country/area, by which users may obtain a rough idea about the surging and dropping time of cases in a specific country/area. A new tab named “SNV Annotation” is appended next to the “Summary Info,” where users can download the full annotation profile or search an individual SNV of interest with the embedded search function. Additionally, the GESS v2 has developed multiple embedded functions for users to quickly extract more information on SNVs and conduct more analyses online, e.g., “Viral Sequence Search,” “Emerging SNVs,” “SNV Enrichment,” and “Lineage Analysis.” The “Viral Sequence Search” allows users to search for SNVs located on interested sequences, given either nucleotides or amino acids or protein names. The “Emerging SNV” explores new SNVs emerging in a specific month under the thresholds chosen by the users. As we have known, the strain/lineage monitoring plays a very important role in public health. The routine analysis of the available genetic sequence data will enable organizations including the Centers for Disease Control and Prevention (CDC) and its public health partners to identify variant viruses for further characterization (13). For example, the B.1.1.7 was first identified as a UK lineage, associated with variants of N501Y, P681H, and numerous other mutations (8). The feature of “Lineage Analysis” in the GESS v2 can help users find lineages enriched in a selected country in regard to their occurrences. Another function entitled “Lineage Migration” shows the dynamic migration paths of a selected lineage over the world within a chosen timeframe on an interactive map. The final feature of “SNV Freq Heatmap” explores the monthly frequencies of SNVs in a heatmap, where the SNVs are clustered based on their dynamic occurrences.

With the power of diverse functions embedded in the database, the GESS v2 can serve as a more comprehensive platform to aid users in analyzing SARS-CoV-2 sequences regarding the distinct transmission and evolution patterns of SNVs and corresponding lineages at different countries/areas during the pandemic period. It also helps monitor newly emerging SNVs at different time points. Diverse enrichment analyses of SNVs and lineages can be conducted directly on the website or performed by users based on the information and results provided by the GESS v2.

## Materials and Methods

### Data Collection

The second version of GESS database contains over 4 million high-quality and high-coverage SARS-CoV-2/hCoV-19 genome sequences downloaded from the GISAID as of January 21, 2022. As the numbers of genome sequence are growing rapidly and today scientists heavily rely on the sequence information to study the evolution of the virus (14), we planned to update our database weekly or biweekly.

### Database Structure

**FIGURE 1:**
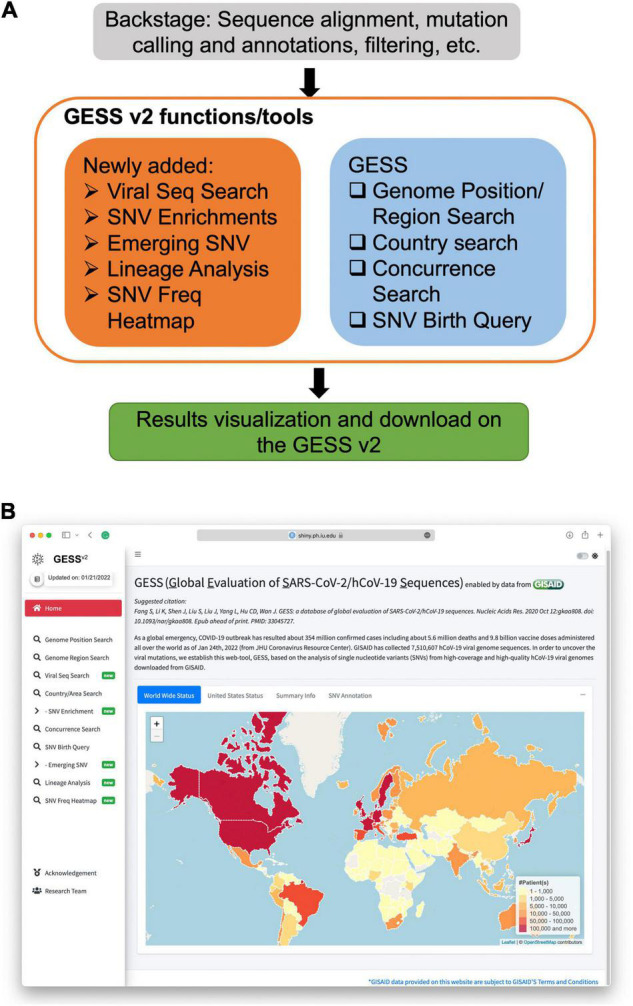
Overview of the global evaluation of SARS-CoV-2/hCoV-19 sequences 2 (GESS v2). **(A)** The GESS v2 functions/tools including the updated ones and those existing in the first version of the GESS. **(B)** Landing page of the GESS v2, where new functions are marked with “new” badges.

The second version of GESS is created with the R programing language and Shiny/ShinyDashboard framework. The main structure and the UI of the landing page are shown in Figure 1.

### Data Preprocessing

The raw complete viral genome sequences of SARS-CoV-2 are collected from the GISAID database and processed as described in Ref. (15). First, the low-quality, low-coverage samples and white spaces in the sequences are removed. Then, the minimap2 (16) is used for the alignment from the sequences to the reference genome Wuhan-Hu-1 (NCBI reference sequence: NC_045512.2). The Wuhan-Hu-1 genome is a full-length viral sequence of SARS-CoV-2, which was discovered in China, December 2019 (17). This reference sequence information derived from Wuhan-Hu-1 provides a basis for almost all SARS-CoV-2 research, especially viral mutation and phylogenetic analysis (18–21) and design of the mainstream vaccines, including BNT162b2 (22), mRNA-1273 (23), and Ad5-nCoV (24). Hence, the Wuhan-Hu-1 is adopted as the reference genome in our study as well. SNVs are annotated based on NC_045512.2 using the ANNOVAR (25).

### Enrichment Analysis

The enrichment analysis is conducted in the “SNV Enrichment” and “Lineage Enrichment” to identify SNVs or lineage significantly enriched in certain country. The statistical significance is calculated based on the cumulative hypergeometric model:


$$\text{p}\,\left(x|M,K,\text{N}\right)=1-\sum_{i=0}^{\text{x}-1}\frac{\left(\begin{array}{c} \text{K}\\ \text{i} \end{array}\right)\,\left(\begin{array}{c} \text{M}-\text{K}\\ \text{N}-\text{i} \end{array}\right)}{\left(\begin{array}{c} \text{M}\\ \text{N} \end{array}\right)}$$

where x and N are the numbers of samples with the SNV/lineage in a specific country and in the world, respectively, whereas K and M are the total number of samples in the desired country and in the world, respectively. All *p*-values are conducted with multiple-test correction using the false discovery rate (FDR) approach without further specifications.

### Identification of Emerging Single-Nucleotide Variant

The emerging SNV in a specific month can be determined by the ratio of the SNV prevalence in the inquired month to that in the previous month. The ratio is named as fold enrichment (F.E.). However, if the number of samples with the SNV is zero in the previous month, it will cause an infinity issue. In that case, we will manually force the number of samples with the SNV as 0.5 instead of zeros during the calculation.

## Results

### Viral Sequence Search

**FIGURE 2:**
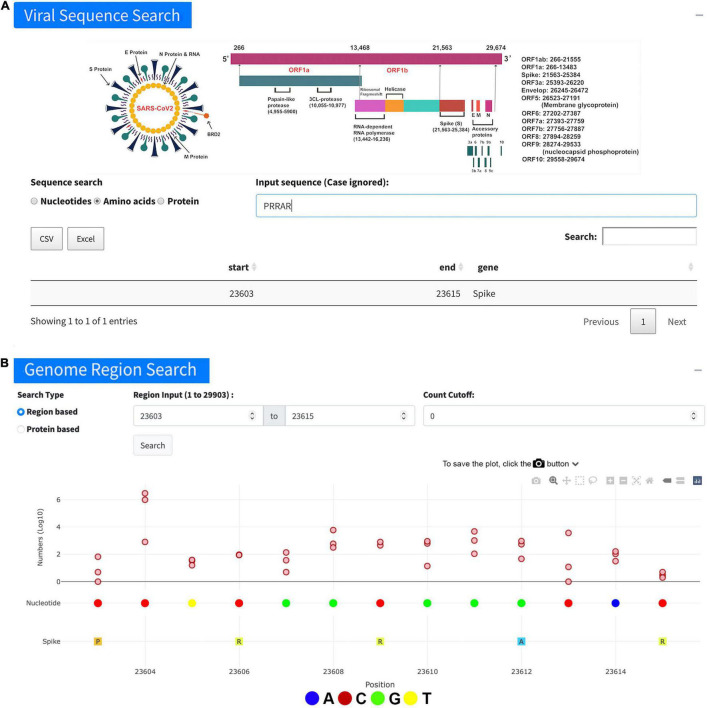
Overview of viral sequence search. **(A)** An example of searching the furin cleavage site “PRRAR” using the “Viral Sequence Search.” The table lists the search results including start and end positions of the sequence. **(B)** More detailed information about the viral genome positions 23,603–23,615 for the sequence “PRRAR” achieved on the page of “Genome region search” after the outcome in the table **(A)** was clicked.

The page of “Viral Sequence Search” contains three subpages, namely, “Nucleotides,” “Amino acids,” and “Proteins.” The “Nucleotides” tab allows users to search SNVs, given a nucleotide sequence. The result is shown in a table with corresponding “start” and “end” positions and the gene regions for the matched sequence. After clicking the region of interest, users will be redirected to the “Genome region search” for further details. The “Amino acids” tab provides a similar function, that is, searching SNVs for amino acid sequences instead of nucleotide sequences. For instance, searching the furin cleavage site PRRAR leads us to the webpage (Figure 2), indicating the number of samples with individual genome sites in this region. If the user is more interested in the protein, the “Protein” search tab will generate a plot to show the mutation numbers along the protein with a table including more detailed information, e.g., all amino acids in the protein, the start position of each, and its corresponding three nucleotides. Clicking on the index of the AA, the user will be redirected to the “Genome region search” for further details. The user can also click on the start position, which will show the “Genome position search” for checking if this position contains any SNV. If it does, the user can further check its distribution on a map with a time series plot.

### Emerging Single-Nucleotide Variant

**FIGURE 3:**
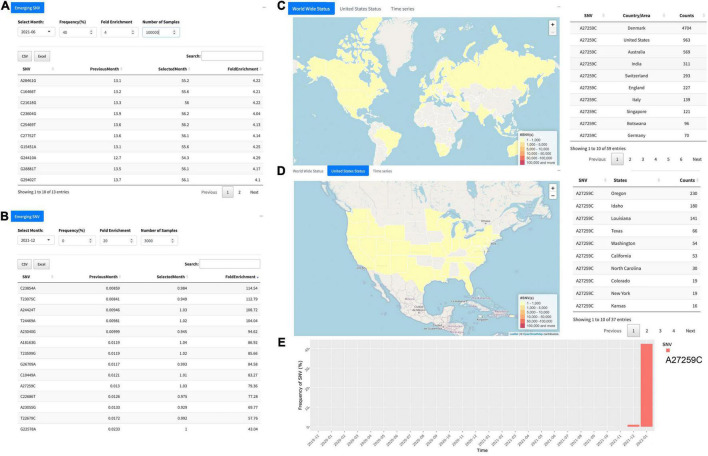
Overview of the function “Emerging SNV.” **(A)** Emerging single-nucleotide variants (SNVs) identified in June 2021 with at least 40% SNV frequency, more than 4-F.E., and at least 1,00,000 samples. It turns out that all the SNVs passing the filter consist of B.1.617.2 (i.e., the delta variant). **(B)** Another example for December 2021 with cutoff of 20-F.E. and more than 3,000 samples harboring the SNV. All 14 SNVs identified, including A27259C, represent the omicron. **(C)** Distribution of A27259C around the world. **(D)** Distribution of the SNV in the United States. **(E)** The temporal pattern of A27259C prevalence, revealing its breakout in December 2021 and significantly a huge increase in January 2022.

The “Emerging SNV” tab unlocks the function to trace or monitor any SNV “emerging” on monthly basis. For example, by selecting “2021-06” in the “Select Month” tab, the user can obtain 13 emerging SNVs in June 2021 if the cutoff values were set as 40 for frequency (%) and 4 for F.E. with minimum 1,00,000 samples carrying the SNVs (Figure 3A). In the result table, each identified SNV is represented in a row, which contains the genome position of the SNV with nucleotides before and after alteration, the frequency of the SNV in the previous month and the selected month, respectively, and its F.E. This allows users to obtain detailed information about the SNVs identified. For the example above (Figure 3A), all these SNVs passing the filters we set are from the B.1.617.2, which is identified as the delta variant, contributing to an enormous number of cases during the past few months (26). With the severe transmission rate around the globe, the group of mutations has mostly occurred in the United Kingdom (B.1.1.7), South Africa (B.1.351), and Brazil (P.1) (27). These changes in the viral genome can vastly affect the diagnostics and even efficacy of the vaccines. Effective surveillance tools for mutations at scientists’ hand can potentially unlock the abilities to improve the existing viral test, treatment, and even vaccines for future variants.

Indeed, the tool of “Emerging SNV” implemented in the GESS v2 can help users in time to monitor individual SNVs or groups of SNVs roaring out of the silence during the past months, which may play a pivotal role in the public health domain. For instance, the tool can be used to uncover new emerging SNVs. In December 2021, 14 SNVs have been detected from more than 3,000 samples with at least 20-F.E. compared to their occurrences in the previous month, November 2021 (Figure 3B). All of them belong to the omicron variants (28), indicating the sudden surge of the omicron since the end of 2021. However, these SNVs can be traced back to different earlier times at different countries/areas during the COVID-19 breakout. For example, 10 out of these 14 SNVs are in S protein, including C22686T (S:S375F) originating in January 2020 in the United States, both A23040G (S:Q493R) and G22578A (S:G339D) in April 2020 in the United Kingdom, T23599G (S:N679K) in April 2020 in France, T22679C (S:S373P) in August 2020 in the United States, C23854A (S:N764K), T23075C (S:Y505H), A24424T (S:Q954H), T24469A (S:N969K), and A23055G (S:Q498R) during the end of 2020 to January of 2021 in the United Kingdom and the United States.

After clicking individual SNV in the table, e.g., A27259C, users are automatically led to the page of “Genome Position Search” for the corresponding SNV, where more information will be popped up (Figures 3C–E). Until present, A27259C has been detected in 4,704 samples collected from Denmark, followed by 963 samples in the United States and 569 in Australia and others (Figure 3C). In the United States, users can see the distribution of SARS-CoV-2 samples carrying A27259C in different states (Figure 3D). The temporal pattern of the SNV has clearly shown the surge of A27259C in December 2021 and a huge increase of its prevalence in January 2022 (Figure 3E).

### Single-Nucleotide Variant Frequency Heatmap

**FIGURE 4:**
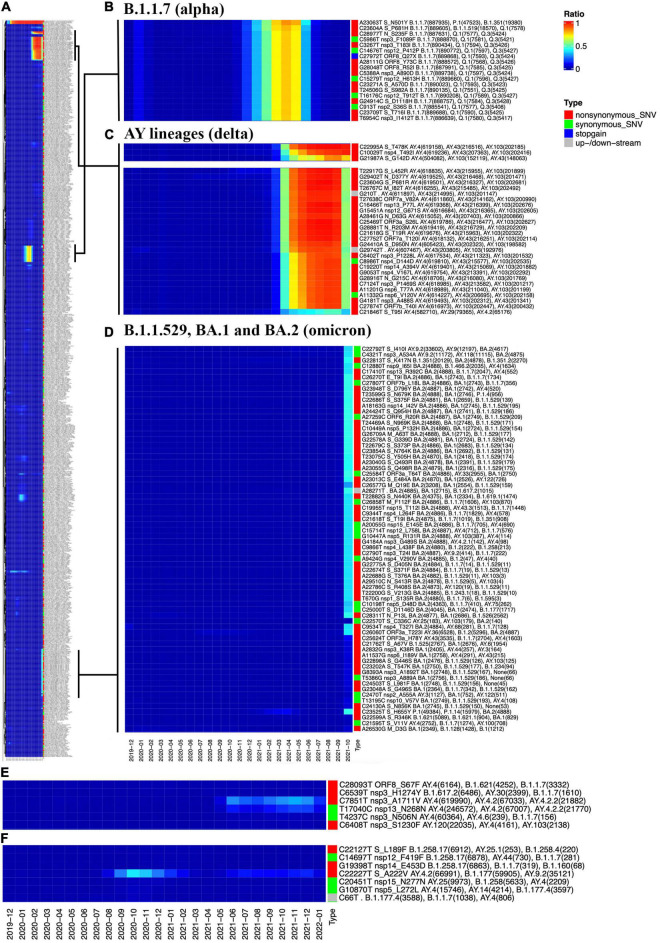
Overview of the “SNV Frequency Heatmap.” **(A)** The full-size heatmap of SNV frequencies as of January 21, 2022. **(B)** The heatmap of B.1.1.7 (i.e., alpha) SNVs. **(C)** The heatmap of AY lineages (i.e., delta) SNVs. **(D)** The heatmap of B.1.1.529 (i.e., omicron) SNVs, including SNVs specific to BA.1 or BA.2. **(E)** The increasing path of a group of SNVs, including C7851T and T17040C from AY.4 and AY.4.2. **(F)** The wave pattern of C22227T, showing that its frequency reached the peak in October and November 2020, then dropped in January and February 2021, followed by a rise again since June 2021.

Another new function of the GESS v2 called as “SNV Freq Heatmap” provides a generally temporal pattern of all SNVs with at least 1% prevalence in terms of the occurrence frequency from December 2019 to the last updated date (Figure 4A). An overview of monthly numbers of all analyzed samples is attached at the top of the heatmap. The SNVs are clustered by their dynamic prevalence and are presented in the heatmap (Figure 4A) by the ComplexHeatmap (29). Users can take advantage of the zoom tool embedded in the website to explore the details about dynamic changes of these SNVs. Three major group variants are zoomed as examples (Figures 4B–D). Almost all individual SNVs composing of the B.1.1.7 (i.e., alpha) and AY lineages (i.e., delta) variants were first detected during the early of 2020 (Figures 4B,C), when they originated independently from different patient samples. After that, these SNVs occurred simultaneously to form diverse viral strains and ruled over during different time periods. For example, B.1.1.7 (i.e., alpha) variant reached its peak around April 2021 (Figure 4B), then decreased, and disappeared when the AY (i.e., delta) outcompeted it. AY (i.e., delta) has become dominant since June 2021 until the end of 2021 (Figure 4C), when the B.1.1.529 (i.e., omicron) outbreak started (Figure 4D). We could clearly see a significant decrease in the majority of delta variants, which dropped down to about 50% in January 2021 (Figure 4C), except a few of them still kept high frequencies (Figure 4C, top panel), including two non-synonymous mutations in the S protein, namely, C22995A and G21987A.

The AY.4 is a sub-variant of the delta and is considered as one of the causes of the fourth wave of the pandemic in Pakistan (30). Two AY.4 representative SNVs, namely, C7851T and T17040C, have shown a trend of rising since July 2021 (Figure 4E). Moreover, C7851T and T17040C share another AY.4.2 variant (some refer this as “Delta plus”) on an increasing trajectory. According to the United Kingdom Health Security Agency,^8^ the delta sub-lineage AY.4.2 (been given the official name VUI-21OCT-01) was designated a Variant Under Investigation (VUI) by the UKHSA in October 2021.

The omicron has two major sub-variants, namely, BA.1 and BA.2, consisting of different sets of SNVs in addition to common ones (Figure 4D). Users can find detailed information about them by utilizing the search tools embedded in the GESS v2. For instance, SNVs G22898A (S:G446S) and A22688G (S:T376A) are observed from omicron BA.1 and BA.2, respectively (Figure 4D). With the function of “Genome position search,” we can see that G22898A has been detected mostly in the United States, followed by Australia, whereas most of the samples bearing A22688G have been identified in Denmark, suggesting the different paths and transmission patterns of these two omicron sub-variants.

In Figure 4F, a non-synonymous mutation on Spike, C22227T (S:A222V), is distinguished from others. The SNV C22227T reached its peak in October and November 2020 as reported (31) and then almost disappeared during the following few months. However, it rose again in July 2021 and kept being active in around 10% of the total population each month. This is in line with other observations (32). The concurrence ratio (C.R.) analysis (11, 15) from the GESS v2 reveals that the SNV has a very low C.R. (<1%) with all alpha representative mutations. However, C22227T presents a high C.R. (about 70%) with many representative SNVs of delta, e.g., T22917G (S:L452R) and C23604G (S:P681R), and a few omicron mutations such as a synonymous mutation on Spike, C22792T (C.R. > 80%), which has been detected mostly in Denmark, England, and other European countries. Although S:A222V was not found associated with significant conformational changes of SARS-CoV-2 S protein as well as aberrant effects on the virus entry (33), the wave pattern of C22227T prevalence and its appearance of co-occurrence with other specific mutations imply a dynamically selective advantage of the SNV as well as uncovered functional roles of S:A222V in viral transmission for special variants.

### Single-Nucleotide Variant Enrichment

**FIGURE 5:**
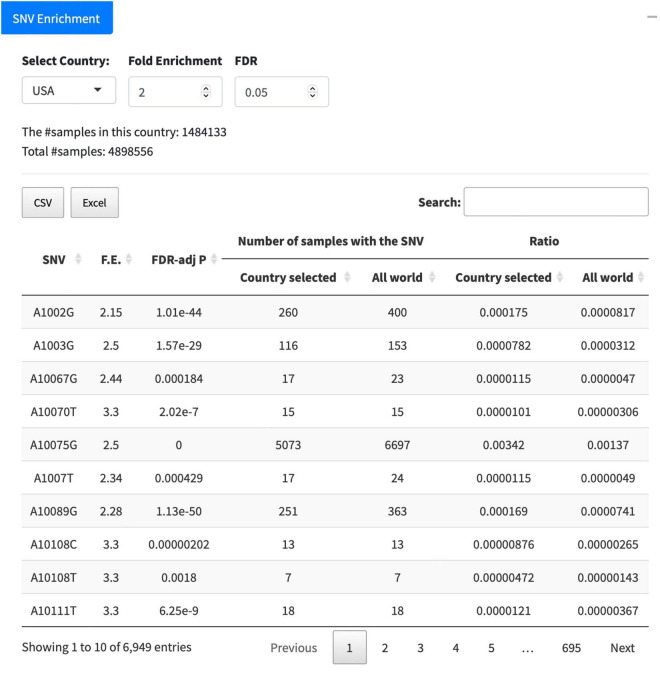
Function of “SNV Enrichment.” The United States is taken as an example to list SNVs significantly [false discovery rate (FDR) < 0.05] enriched (F.E. > 2) in the country.

The tab of “Country/Area Search” lists all SNVs detected from the samples collected in a selected country. After downloading the numbers of samples carrying corresponding SNVs in the country for each month, users are able to perform the enrichment analysis to identify surging SNVs in this country within a specific time period of their interest, e.g., from February 2021 to June 2021. Another new function “SNV Enrichment” is implemented in the GESS v2 as well. It unveils SNVs significantly enriched in a selected country compared with those in all countries. Taking the United States as an example, we can see 6,949 SNVs with significantly (FDR < 0.05) higher (at least 2-F.E.) prevalence in the United States compared with that in all countries (Figure 5). The outcome table lists the detailed information for SNVs significantly overrepresented in the country, including SNV information, F.E., and FDR-adj *p*-values, together with other more details, e.g., number of samples with the SNV in this country or in the world, and ratios. The results can be downloaded in the format of either csv or excel.

### Lineage Analysis

**FIGURE 6:**
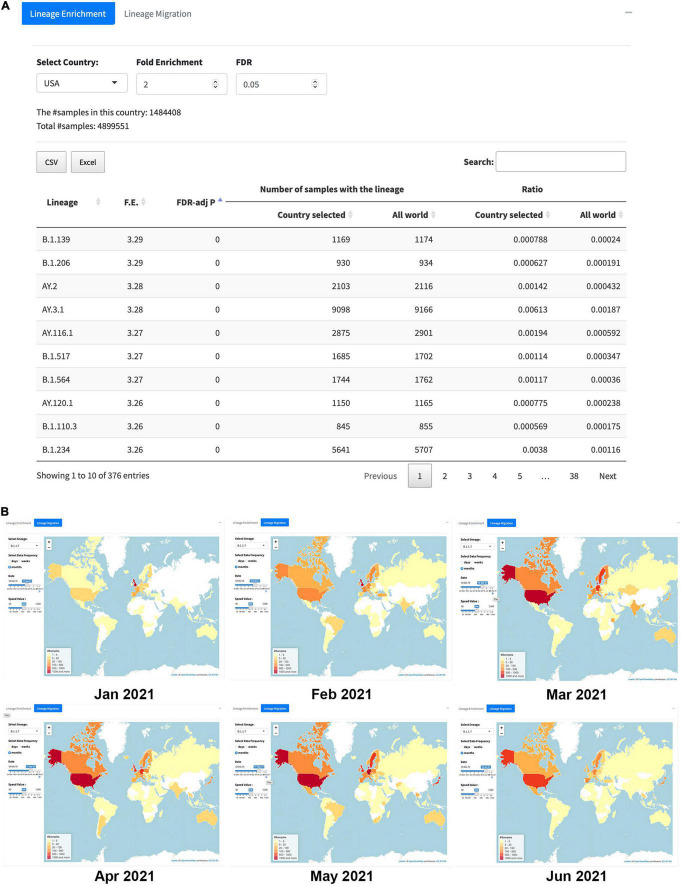
The overall look of the “Lineage Analysis.” **(A)** An example of “Lineage Enrichment” results in the United States with the cutoffs, F.E. > 2, and FDR < 0.05. **(B)** “Lineage Migration” patterns for B.1.1.7 (i.e., alpha); six key frames correspond to the time from January 2021 to June 2021 captured from the animated lineage migration map, indicating that the variant spread to the world from January to May 2021, then started to disappear since June 2021.

The section “Lineage Analysis” includes two functions, namely, “Lineage Enrichment” and “Lineage Migration.” The “Lineage Enrichment” allows users to identify lineages instead of individual SNVs significantly overrepresented in the selected country. The outcome table is similar to what we see from the “SNV Enrichment” analysis, including the information about the lineage, F.E., FDR-adj *p*-value, number of samples with the lineage in the selected country or in the world, and the ratios in the country and the world, respectively (Figure 6A).

The second tab for the section “Lineage Analysis” is the “Lineage Migration.” In this tab, users can choose lineages of their interests from top 50 lineages to check their dynamic transmission pattern on the map, which exhibits where and when the lineage firstly identified and how the lineage spread to the world. Figure 6B contains six key frames captured from the animated lineage migration maps of the B.1.1.7 (i.e., alpha) variant from January to June 2021. It clearly shows that the B.1.1.7 was first detected from a sample collected in September 2020 and then outbroke in the United Kingdom in January 2021. After that, it has kept increasing vastly in the United States for 3 months (from February to May 2021), and then the alpha variant started to decrease since June 2021.

## Discussion

The first version of the GESS (11) provided several useful and efficient tools for users to search individual SNVs of their interests. The GESS v2 enhances the power in many ways to provide users with diverse detailed information about SARS-CoV-2 sequences, SNVs, and lineage information intuitively by implementing more comprehensive novel features with convenient clicking and choosing operations. With magnified capacities of the multiple search functions and over 4 million samples achieved from the GISAID as of January 21, 2022, the GESS v2 brings users multiple approaches to search viral sequences and check if a lineage/SNV is enriched in the interested countries. Users can see how an interesting lineage has spread among countries on the interactive map. The highlighted functions of the GESS v2 are “Emerging SNV” and “SNV Freq Heatmap,” which provide a straightforward toolset to allow researchers to check the emerging mutations in time. These tools can monitor or even predict new SARS-CoV-2 variants, which will become potentially dominant after an incubation period. The updated database with vigorous approaches helps people understand how the SARS-CoV-2 has been evolving and adjust diagnostic tests to prevent the variants from evading detection. The sequence conservation information from over millions of SARS-CoV-2 genomes also has the power to assist scientists in the design of new vaccines against emerging dominant variants.

## Data Availability Statement

The data analyzed in this study is subject to the following licenses/restrictions: The release of original data must go through the GISAID based on the agreements. All analyzed results can be found and downloaded from the website, https://shiny.ph.iu.edu/GESS_v2/. Requests to access these datasets should be directed to GESS v2, https://shiny.ph.iu.edu/GESS_v2/.

## Author Contributions

JS, LY, C-DH, KY, and JW initiated and designed the studies. KL, AW, SL, SF, AL, and JW conducted analyses on sequence alignment, annotation, and developed new functions and tools for the database. KL, AW, and SL worked on reorganization, development, and updates of the website. KY and JW supervised the whole study and provided guidance. KL, JS, KY, and JW wrote the manuscript. All authors reviewed/finalized the manuscript and approved the submission of the article.

## Conflict of Interest

The authors declare that the research was conducted in the absence of any commercial or financial relationships that could be construed as a potential conflict of interest.

## Publisher’s Note

All claims expressed in this article are solely those of the authors and do not necessarily represent those of their affiliated organizations, or those of the publisher, the editors and the reviewers. Any product that may be evaluated in this article, or claim that may be made by its manufacturer, is not guaranteed or endorsed by the publisher.

## Footnotes

1
https://www.gisaid.org/

2
https://covdb.stanford.edu/

3
https://outbreak.info

4
http://nextstrain.org/

5
http://cov-lineages.org/

6
http://cov-glue.cvr.gla.ac.uk/#/home

7
http://covidep.ust.hk/

8
https://www.gov.uk/government/news/covid-19-variants-identified-in-the-uk
